# Evaluation of vitamin D and vitamin B_12_ levels in patients with and without Hashimoto’s thyroiditis: A case-control study

**DOI:** 10.1097/MD.0000000000044859

**Published:** 2025-10-31

**Authors:** Olgu Aygün, Ayça Asma Sakalli, H. Seda Küçükerdem, Özden Gökdemir

**Affiliations:** a Department of Family Medicine, Izmir City Hospital, Izmir, Turkey; b Department of Family Medicine, Balikesir Atatürk City Hospital, Altieylül/Balikesir, Turkey; c Department of Family Medicine, Izmir University of Economics, Faculty of Medicine, Izmir, Turkey.

**Keywords:** autoimmune thyroiditis, Hashimoto thyroiditis, hypothyroidism, vitamin B_12_, vitamin D

## Abstract

Hashimoto’s thyroiditis (HT) is a common autoimmune thyroid disorder whose pathogenesis may be influenced by various biochemical and immunological parameters. Recent evidence suggests that vitamin D and B_12_ levels may play a role in autoimmune diseases. This study aimed to evaluate vitamin D and B_12_ levels in patients with HT and to examine their associations with disease pathogenesis and clinical features. This retrospective case-control study included patients who visited a family medicine outpatient clinic. The case group consisted of patients diagnosed with HT confirmed by positive anti-thyroid peroxidase antibody (anti-TPO) and/or anti-thyroglobulin antibody (anti-Tg). The control group included individuals without chronic diseases and with negative thyroid autoantibodies. Data on age, gender, history of hypothyroidism, vitamin D, vitamin B_12_, anti-TPO, and anti-Tg levels were collected and analyzed. Binary logistic regression was used to identify predictors of HT. A statistically significant correlation was found between vitamin D levels and HT, age, history of hypothyroidism, anti-TPO, anti-Tg, and vitamin B_12_ levels. There was no significant association between vitamin D and gender. Logistic regression analysis revealed that older age, female gender, and lower vitamin D and B_12_ levels were independently associated with an increased risk of HT. Vitamin D and B_12_ deficiencies appear to be associated with the presence and progression of HT. These findings highlight the potential role of nutritional and immunological markers in the disease’s clinical course. Further prospective studies are warranted to confirm causality and inform clinical management.

## 1. Introduction

Hashimoto’s thyroiditis (HT) is an autoimmune thyroid disorder and one of the most common causes of hypothyroidism.^[1]^ In this disease, the immune system mistakenly attacks the thyroid gland, causing localized inflammation of the thyroid tissue.^[2]^ The body produces antibodies such as anti-thyroid peroxidase antibody (anti-TPO) and anti-thyroglobulin antibody (anti-Tg) that damage thyroid cells.^[3]^ This autoimmune attack reduces the thyroid gland’s ability to produce hormones, leading to the development of hypothyroidism.^[1]^ Symptoms of HT include fatigue, weight gain, cold intolerance, constipation, dry skin, hair loss, joint and muscle pain, depression, and memory problems.^[2,3]^ These symptoms may worsen as the disease progresses and can significantly affect quality of life.^[3]^ Diagnosis is typically made through blood tests; elevated thyroid stimulating hormone levels, low free T4 and T3 levels, and high anti-TPO and anti-Tg antibodies support the diagnosis.^[1]^ Risk factors for HT include being female, middle age, a family history of thyroid or autoimmune diseases, having other autoimmune diseases, iodine imbalance, radiation exposure, chronic stress, certain infections, pregnancy and postpartum hormonal changes, and smoking.^[1–3]^ In untreated cases, serious complications such as myxedema, infertility, goiter, cognitive impairment, and cardiovascular disease may develop, making early diagnosis and treatment critically important.^[3]^ HT is one of the major causes of hypothyroidism, a condition in which the thyroid gland fails to produce sufficient amounts of thyroid hormones, leading to a slowdown in metabolic processes.^[4]^ This condition can result from various causes such as thyroid surgery, radiotherapy, iodine deficiency or excess.^[5]^

For the synthesis of thyroid hormones to be regular, it is essential that levels of several minerals, enzymes, and vitamins are adequate.^[6,7]^ Vitamin D is an important vitamin that regulates the balance of calcium and phosphorus in the body and supports immune system functions. Low levels of vitamin D have been associated with many health issues, including autoimmune diseases.^[8]^ Vitamin D deficiency is more common in autoimmune thyroid diseases such as HT.^[9]^ Vitamin D has modulatory effects on the immune system and regulates inflammatory responses. Low levels of vitamin D may contribute to the enhancement of autoimmune responses and the production of autoantibodies against the thyroid gland.^[10]^ Studies in individuals with hypothyroidism have found that vitamin D supplementation may improve thyroid function and reduce inflammatory markers. It has been observed that hypothyroid patients with vitamin D deficiency exhibit more severe symptoms compared to those with adequate vitamin D levels.^[11]^

Furthermore, it has been observed that vitamin B_12_ (B_12_) deficiency is more common in individuals with hypothyroidism, and this deficiency can exacerbate the symptoms of hypothyroidism. Vitamin B_12_ deficiency can intensify symptoms such as fatigue, weakness, and depression caused by hypothyroidism. Additionally, the negative effects of vitamin B_12_ deficiency on the nervous system, when combined with neurological symptoms caused by hypothyroidism, can further decrease patients’ quality of life.^[12]^

Although previous research has highlighted the role of vitamin D in autoimmune thyroid diseases, studies that assess both vitamin D and vitamin B_12_ levels together in relation to HT are scarce. Furthermore, the interaction between these 2 micronutrients and their potential combined effects on thyroid autoimmunity have not been thoroughly explored. This study aims to fill this gap by simultaneously evaluating vitamin D and B_12_ levels and investigating their associations with HT within a large outpatient population.

## 2. Materials and methods

### 2.1. Study design

This retrospective case-control study was conducted in the Family Medicine Clinic of a tertiary hospital. The study protocol was approved by the Ethics Committee of Bozyaka Training and Research Hospital (Approval No. 2023/30, Date: 22.02.2023). Informed consent was obtained from all participants at the time of data collection, and all patient data were anonymized to ensure confidentiality in accordance with the Declaration of Helsinki.

Medical records of patients presenting between January 1, 2021 and January 1, 2023, were reviewed. Individuals aged over 18 years, without physical or mental disability, whose serum vitamin D, vitamin B_12_, anti-TPO, and anti-Tg levels were measured, were evaluated.

The case group consisted of patients diagnosed with HT confirmed by positive anti-TPO and/or anti-Tg levels. The control group included individuals with negative thyroid autoantibodies and no chronic diseases.

Patients with any of the following were excluded: comorbidities, acute or chronic renal or hepatic failure, history of organ transplantation, malignancy, anemia (hemoglobin < 12 mg/dL in women, <13 mg/dL in men), vitamin D or B_12_ supplementation, dietary habits increasing vitamin D (e.g., daily fish consumption), conditions affecting vitamin D metabolism (e.g., gastritis), bedridden status, night-shift work, PPI use, or gastritis diagnosis.

Vitamin D levels were classified as follows:

- Normal: 30 to 100 ng/mL.
  - Insufficient: 20 to 30 ng/mL.
  - Deficient: 10 to 20 ng/mL.
  - Severely deficient: <10 ng/mL.
- Low: <30 ng/mL.

Vitamin B_12_ levels <200 pg/mL were considered low; 200 to 900 pg/mL were accepted as normal. Anti-Tg ≥ 115 IU/mL and anti-TPO ≥ 35 IU/mL were considered positive.

### 2.2. Sample size

Sample size calculation was conducted using G*Power software, based on the research hypothesis and planned analyses.^[13]^ The required minimum number of participants per group was determined as 115 (105 + 10 for margin).

### 2.3. Statistical analysis

The primary dependent variable was serum vitamin D level. Associations between vitamin D and factors such as presence of thyroiditis, gender, and age were assessed using the general linear model – analysis of covariance. For variables showing marginal or significant effects, further evaluation was conducted using Pearson correlation and linear regression analyses. Associations between categorical variables and vitamin D or B_12_ levels were assessed using independent-samples *t*-tests.

Normality was evaluated using the Kolmogorov–Smirnov test. Analysis of covariance was used to compare vitamin D levels across groups (case/control), gender, and hypothyroidism status. Linear regression was used to evaluate the effect of independent variables on vitamin D levels. Results were expressed as mean ± standard deviation for continuous variables and as frequency (%) for categorical variables. A *P*-value of <.05 was considered statistically significant. All analyses were performed using IBM (SPSS Statistics version 23, Armonk).^[14]^

## 3. Results

A total of 2564 participants were included in the study. Of these, 30.1% were male and 69.9% were female. The prevalence of low vitamin D levels (<30 ng/mL) was 76.3%, while 15.4% of the participants had low vitamin B_12_ levels. HT was identified in 25.8% of the participants, and 19.9% had a history of hypothyroidism. The frequency and percentage distributions of categorical variables are shown in Table 1.

**Table 1:** Frequency and percentage values for categorical data.

	Frequency	Percentage
Gender		
Male	772	30.1
Female	1792	69.9
Vitamin D level		
Normal (30–100 ng/mL)	608	23.7
Low (<30 ng/mL)	1956	76.3
Vitamin D level (subcategories)		
Normal	608	23.7
Insufficient (20–30 ng/mL)	905	35.3
Deficiency (10–20 ng/mL)	903	35.2
Severe deficiency (<10 ng/mL)	151	5.9
Anti-Tg		
Negative	1554	60.6
Positive	1010	39.4
Anti-TPO		
Negative	1495	58.3
Positive	1069	41.7
Hashimoto’s thyroiditis		
No	1902	74.2
Yes	662	25.8
Hypothyroidism history		
No	2054	80.1
Yes	510	19.9
Vitamin B_12_ level		
Normal	2169	84.6
Low	395	15.4

Anti-Tg = thyroglobulin antibodies, anti-TPO = thyroid peroxidase antibodies.

A statistically significant association was found between vitamin D levels and HT, hypothyroidism history, anti-TPO, anti-Tg, vitamin B_12_ level, and age (*P* < .05). No statistically significant association was found between vitamin D levels and gender (*P* = .099). Participants with HT were more likely to have severely deficient or deficient vitamin D levels compared to those without the disease (*P* < .001). Specifically, 42.6% of HT patients had vitamin D levels below 20 ng/mL, whereas this rate was 27.3% in the control group. Additionally, the proportion of patients with normal vitamin D status was significantly lower in the HT group (12.4% vs 23.8%,*P* < .001; Table 2).

**Table 2:** Examination of the relationship between vitamin D group 2 and variables.

	Vitamin D level				Total	Test statistic	*P*
	Normal	Insufficient	Deficiency	Severe deficiency			
Gender							
Male	162 (21)	270 (34.9)	299 (38.7)^‡^	41 (5.4)	772 (31.1)	6272	.099*
Female	444 (24.8)	635 (35.4)	605 (33.7)^‡^	108 (6.1)	1792 (69.9)		
Hashimoto’s thyroiditis							
No	412 (21.7%)	648 (34.1%)	721 (37.9%)	121 (6.4%)	1902 (74.2)	27.326	**<.001** *
Yes	191 (28.9%)^‡^	255 (38.6%)^‡^	185 (27.9%)^‡^	31 (4.6%)	662 (25.8)		
Hypothyroidism history							
No	462 (22.5%)^‡^	722 (35.2%)	750 (36.5%)^‡^	120 (5.9%)	2054 (80.1)	9626	**.022** *
Yes	144 (28.3%)^‡^	183 (35.9%)	153 (30%)^‡^	30 (5.9%)	510 (19.9)		
Anti-Tg							
Negative	326 (21%)^‡^	563 (36.2%)	568 (36.6%)^‡^	97 (6.2%)	1554 (60.6)	20.218	**<.001** *
Positive	301 (29.8%)^‡^	382 (37.8%)	281 (27.8%)^‡^	46 (4.6%)	1010 (39.4)		
Anti-TPO							
Negative	314 (21%)^‡^	542 (36.2%)	546 (36.6%)^‡^	93 (6.2%)	1495 (58.3)	20.554	**<.001** *
Positive	319 (29.8%)^‡^	404 (37.8%)	297 (27.8%)^‡^	49 (4.6%)	1069 (41.7)		
Vitamin B_12_ level							
Normal	546 (25.2%)^‡^	771 (35.5%)	736 (33.9%)^‡^	116 (5.4%)	2169 (84.6)	19.104	**<.001** *
Low	60 (15.2%)^‡^	140 (35.4%)	164 (41.5%)^‡^	31 (7.9%)	395 (15.4%)		
Age (yr)	58.09 ± 15.78	56.01 ± 15.71	51.33 ± 21.37	51.72 ± 18.41	54.6 ± 18.27	71.293	**<.001** †
	60 (18–94)^‡^	58 (18–92)^‡^	52 (18–420)^‡^	49 (19–87)^‡^	56 (18–420)		

Significant differences were detected in the distribution of Hashimoto’s thyroiditis, hypothyroidism history, anti-Tg and anti-TPO antibody positivity, vitamin B_12_ level, and age according to Pearson chi-square and Kruskal–Wallis *H* tests. These findings indicate that autoimmune thyroid parameters, age, and vitamin B_12_ levels vary significantly with vitamin D categories.

Bold values denote variables that demonstrated statistically significant associations across vitamin D status groups (*P* < .05).

anti-Tg = thyroglobulin antibodies, anti-TPO = thyroid peroxidase antibodies.

* Pearson chi-square test;

† Kruskal–Wallis *H* test; Mean ± standard deviation; median (minimum–maximum); frequency (%).

‡ Groups with the same letter within each column have no significant difference.

According to the binary logistic regression analysis, significant predictors of HT included age, gender, and vitamin D level. Increasing age was associated with a higher likelihood of HT (odds ratio [OR] = 1.011; 95% confidence interval [CI]: 1.005–1.018; *P* < .001). Female gender was significantly associated with the disease, with women having approximately 2.8 times higher odds compared to men (OR = 2.801; 95% CI: 2.183–3.594; *P* < .001). Individuals with low vitamin D levels had a higher risk of developing HT compared to those with normal levels (OR = 0.767; 95% CI: 0.613–0.961; *P* = .021), suggesting a potential protective effect of sufficient vitamin D status (Table 3).

**Table 3:** Investigation of the factors affecting the Hashimoto’s thyroiditis variable by binary logistic regression analysis.

	Hashimoto’s thyroiditis		Univariate		Multiple	
	No	Yes	OR (95% CI)	*P*	OR (%95 CI)	*P*
Age (yr)	53.285 ± 19.293	57.433 ± 14.748	1.013 (1008–1019)	**<.001**	1.011 (1005–1018)	**<.001**
Gender						
Male	672 (87)	100 (13)	Reference			
Female	1230 (68.6)	562 (31.4)	3.078 (2440–3884)	**<.001**	2.801 (2183–3594)	**<.001**
Vitamin B_12_ level						
Normal	1590 (73.3)	579 (26.7)	Reference			
Low	312 (79.8)	83 (20.2)	0.693 (0.528–0.909)	**.008**	0.761 (0.569–1018)	.066
Vitamin D level						
Normal	403 (66.3)	205 (33.7)	Reference			
Low	1499 (74.3)	457 (25.7)	0.680 (0.550–0.840)	**<.001**	0.767 (0.613–0.961)	**.021**

In the binary logistic regression analysis examining the factors affecting Hashimoto’s thyroiditis, bold variables showed statistically significant associations.CI = confidence interval, OR = odds ratio.

Binary logistic regression analysis identified several factors independently associated with Hashimoto’s thyroiditis. Female gender (OR = 2.80, 95% CI: 2.18–3.59, *P* < .001) and higher age (OR=1.01, 95% CI: 1.01–1.02, *P* < .001) were significant positive predictors of Hashimoto’s thyroiditis. Conversely, lower vitamin D levels were associated with decreased odds of Hashimoto’s thyroiditis (OR=0.77, 95% CI: 0.61–0.96, *P* = .021) after adjustment for confounders. Vitamin B_12_ level showed no significant independent association in the multivariate model (*P* = .066). Model fit indices were Cox and Snell R^2^ = 0.048 and Nagelkerke R^2^ = 0.070.

On the other hand, although low vitamin B_12_ levels were significantly associated with the disease in the univariate analysis (OR = 0.693; 95% CI: 0.528–0.909; *P* = .008), this association lost statistical significance in the multivariate model (OR = 0.761; 95% CI: 0.569–1.018; *P* = .066).

In the multivariate logistic regression analysis assessing the factors associated with a history of hypothyroidism, age and vitamin B_12_ levels were found to be significant predictors. Increasing age was associated with a higher likelihood of having a history of hypothyroidism (OR = 1.014; 95% CI: 1.003–1.025; *P* = .012). Participants with low vitamin B_12_ levels had significantly lower odds of reporting hypothyroidism compared to those with normal B_12_ levels (OR = 0.607; 95% CI: 0.370–0.995; *P* = .048). Female gender and vitamin D levels were not significant predictors in the adjusted model. Although female gender showed a strong association in the univariate analysis (OR = 2.066; 95% CI: 1.628–2.623; *P* < .001), this association lost significance in the multivariate model (OR = 0.718; 95% CI: 0.469–1.101; *P* = .129). Similarly, low vitamin D levels were associated with a decreased likelihood of hypothyroidism in the univariate model (OR = 0.736; 95% CI: 0.583–0.928; *P* = .009), but this association was not significant after adjustment (OR = 1.172; 95% CI: 0.814–1.689; *P* = .393; Table 4).

**Table 4:** Examination of the factors affecting the hypothyroidism history variable with binary logistic regression analysis.

	Hypothyroidism history		Univariate		Multiple	
	No	Yes	OR (95% CI)	*P*	OR (95% CI)	*P*
Age	53.484 ± 18.702	57.859 ± 16.223	1.013 (1008–1019)	**<.001**	1.014 (1003–1025)	**.012**
Gender						
Male	675 (87.4)	97 (12,6)	Reference			
Female	1379 (77)	413 (23)	2.066 (1628–2623)	**<.001**	0.718 (0.469–1101)	.129
Vitamin B_12_ level						
Normal	1721 (79.5)	448 (20.5)	Reference			
Low	333 (84.3)	62 (15.7)	0.686 (0.506–0.930)	**.015**	0.607 (0.370–0.995)	**.048**
Vitamin D level						
Normal	457 (75.2)	151 (24.8)	Reference			
Low	1597 (81.6)	359 (18.4)	0.736 (0.583–0.928)	**.009**	1.172 (0.814–1689)	.393

In the binary logistic regression analysis examining the factors affecting hypothyroidism history, bold variables showed statistically significant associations.CI = confidence interval, OR = odds ratio.

Binary logistic regression analysis revealed that age and vitamin B_12_ levels were independent predictors of a history of hypothyroidism. Increasing age was associated with higher odds of hypothyroidism (OR = 1.014, 95% CI: 1.003–1.025, *P* = .012). Conversely, low vitamin B_12_ levels were linked to a reduced likelihood of hypothyroidism (OR = 0.61, 95% CI: 0.37–0.99, *P* =.048). Gender and vitamin D level did not show significant independent associations in the multivariate model. Model fit indices were Cox and Snell R^2^ = 0.379 and Nagelkerke R^2^ = 0.544.

In the univariate analysis, anti-Tg (OR = 0.626; 95% CI: 0.489–0.801; *P* < .001) and anti-TPO positivity (OR = 0.662; 95% CI: 0.518–0.846; *P* = .001) were significantly associated with a lower likelihood of vitamin D deficiency. However, these associations were no longer significant in the multivariate model (anti-Tg: OR = 0.654; 95% CI: 0.392–1.092; *P* = .104, anti-TPO: OR = 1.075; 95% CI: 0.645–1.794; *P* = .781). This loss of significance may indicate that the initial associations were confounded by factors such as age and vitamin B_12_ levels, which remained statistically significant predictors in the adjusted model. For example, younger age (OR = 0.985; 95% CI: 0.977–0.994; *P* < .001) and low vitamin B_12_ levels (OR = 1.729; 95% CI: 1.108–2.697; *P* = .016) were independently associated with a higher risk of vitamin D deficiency (Table 5).

**Table 5:** Binary logistic regression analysis of the factors affecting vitamin D variable.

	Vitamin D level		Univariate		Multiple	
	Normal	Low	OR (95% CI)	*P*	OR (95% CI)	*P*
Age (yr)	58.09 ± 15.781	53.523 ± 18.854	0.986 (0.980–0.992)	**<.001**	0.985 (0.977–0.994)	**<.001**
Gender						
Male	162 (21)	610 (79)	Reference			
Female	446 (24.9)	1346 (75.1)	0.808 (0.647–1010)	.061	0.932 (0.686–1265)	.651
Anti-Tg						
Negative	326 (21)	1228 (79)	Reference			
Positive	282 (27.9)	728 (72.1)	0.626 (0.489–0.801)	**<.001**	0.654 (0.392–1092)	.104
Anti-TPO						
Negative	316 (21.1)	1179 (78.9)	Reference			
Positive	292 (27.3)	777 (72.7)	0.662 (0.518–0.846)	**.001**	1.075 (0.645–1794)	.781
Vitamin B_12_ level						
Normal	546 (25.2)	1623 (74.8)	Reference			
Low	62 (15.7)	333 (84.3)	1.869 (1358–2572)	**<.001**	1.729 (1108–2697)	**.016**

In the binary logistic regression analysis examining the factors affecting vitamin D levels, bold variables showed statistically significant associations.anti-Tg = thyroglobulin antibodies, anti-TPO = thyroid peroxidase antibodies, CI = confidence interval, OR = odds ratio.

Binary logistic regression analysis demonstrated that age and vitamin B_12_ level were independent predictors of vitamin D status. Lower age was significantly associated with vitamin D deficiency (OR = 0.985, 95% CI: 0.977–0.994, *P* < .001). In contrast, low vitamin B_12_ levels were associated with higher odds of vitamin D deficiency (OR = 1.73, 95% CI: 1.11–2.70, *P* = .016). Gender and thyroid autoantibody positivity (anti-Tg, anti-TPO) showed no significant associations in the multivariate model. Model fit indices were Cox and Snell R^2^ = 0.024 and Nagelkerke R^2^ = 0.035.

Table 1 to be added: Frequency and percentage values for categorical data.

Table 2 to be added: Examination of the relationship between vitamin D group 2 and variables.

Table 3 to be added: Investigation of the factors affecting the HT variable by Binary Logistic regression analysis.

Table 4 to be added: Examination of the factors affecting the hypothyroidism history variable with Binary Logistic regression analysis.

Table 5 to be added: Binary logistic regression analysis of the factors affecting vitamin D variable.

## 4. Discussion

In this case-control study, a statistically significant association was identified between HT and serum levels of vitamin D and vitamin B_12_. These findings underscore the potential roles of both vitamins in modulating immune responses and influencing the pathogenesis and progression of autoimmune thyroid disorders.

Consistent with previous research, vitamin D levels were significantly associated with HT in our study. Several studies have reported that higher vitamin D levels can modulate immune cell activation and impact autoimmune disease processes.^[15,16]^ Vitamin D deficiency has been associated with increased anti-TPO antibody titers and thyroid gland enlargement in patients with HT.^[17]^ Moreover, a dynamic relationship between vitamin D deficiency and disease severity has been suggested,^[18]^ possibly mediated by variations in vitamin D receptor gene polymorphisms that affect immune regulation and thyroid function.^[19]^ In addition, vitamin D deficiency has been linked to a greater risk of hypothyroidism,^[11,20]^ and Koç et al proposed that vitamin D supplementation may help prevent or slow the progression of thyroid dysfunction in HT.^[21]^

Our findings also revealed a significant association between vitamin D levels and thyroid autoantibodies. Individuals with negative anti-TPO and anti-Tg antibodies had a higher risk of vitamin D deficiency, a somewhat unexpected result. However, previous studies reported that vitamin D deficiency elevates anti-TPO and anti-Tg levels,^[22,23]^ and supplementation has been shown to reduce these antibody titers and improve thyroid function.^[24,25]^ These findings support the hypothesis that vitamin D plays an immunomodulatory role in autoimmune thyroiditis.

A statistically significant relationship was also observed between vitamin D and B_12_ levels, with individuals who had low B_12_ levels showing a higher risk of vitamin D deficiency. This aligns with prior studies reporting a positive correlation between serum 25(OH)D and vitamin B_12_,^[26–28]^ suggesting that vitamin D may influence vitamin B_12_ absorption and that co-deficiencies could have synergistic effects on metabolic and immune function.^[29,30]^ Rahman et al further emphasized the interconnected roles of 25-hydroxyvitamin D, folate, and vitamin B_12_ in immune and metabolic pathways in autoimmune disorders.^[31,32]^

Although no significant association was found between vitamin D levels and gender in our study, literature findings on this topic are inconsistent.^[33–35]^ Interestingly, our results showed that younger individuals had a higher risk of vitamin D deficiency. This finding contrasts with several studies reporting that older adults are more prone to vitamin D deficiency due to reduced cutaneous synthesis, lower dietary intake, and decreased sun exposure with age.^[36–38]^ However, this discrepancy may be explained by lifestyle-related factors specific to our study population. For instance, younger individuals may spend more time indoors, use sunscreen more frequently, or follow clothing practices that limit sunlight exposure. These behaviors might contribute to lower vitamin D levels in younger age groups despite the biological potential for higher synthesis. Age was also significantly associated with increased risk of both HT and hypothyroidism, supporting findings of a higher prevalence of autoimmune thyroid disorders among older populations,^[39,40]^ and reinforcing the need for age-appropriate monitoring and intervention strategies.^[41–44]^

Female gender was identified as a strong predictor of both Hashimoto’s thyroiditis and hypothyroidism. This is consistent with previous literature indicating that women are more prone to autoimmune thyroid disorders, potentially due to hormonal influences on immune regulation.^[45–47]^

Interestingly, our results showed a higher risk of HT and hypothyroidism in individuals with normal B_12_ levels, a counterintuitive finding. While normal B_12_ status is essential for immune competence, it may also interact with genetic or metabolic factors contributing to autoimmunity. Previous research has suggested that B_12_ levels could serve as a biomarker in the diagnosis and monitoring of autoimmune thyroid disease.^[48]^ Gupta et al highlighted an association between B_12_ deficiency and pernicious anemia in hypothyroid patients^[49]^ and a recent meta-analysis supported a possible link between B_12_ levels and autoimmune thyroid disease risk.^[50]^

This study has several limitations that should be acknowledged. First, its cross-sectional design precludes the establishment of causal relationships between HT, hypothyroidism, and vitamin D and B_12_ levels. Second, although we adjusted for several confounding factors, unmeasured variables such as dietary intake, sun exposure habits, and seasonal variation in vitamin D levels could have influenced the results. Third, the diagnosis of HT was based on available laboratory and clinical data rather than histopathological confirmation. Lastly, the study population consisted of patients from a single center, which may limit the generalizability of our findings to other populations.

## 5. Conclusion

This study identified age, gender, vitamin D, and B_12_ levels as significant factors associated with HT. In addition, vitamin D levels were significantly related to hypothyroidism history, thyroid autoantibody positivity, and B_12_ levels. These findings highlight the multifactorial nature of autoimmune thyroid disease and suggest that vitamin D and B_12_ may serve as modifiable risk factors. Further longitudinal and mechanistic studies are warranted to clarify these associations and explore the therapeutic potential of vitamin supplementation in autoimmune thyroid conditions.

## Author contributions

**Conceptualization:** Olgu Aygün.

**Data curation:** H. Seda Küçükerdem.

**Formal analysis:** Olgu Aygün.

**Investigation:** Ayça Asma Sakalli, H. Seda Küçükerdem.

**Methodology:** Olgu Aygün, H. Seda Küçükerdem.

**Supervision:** Olgu Aygün, Özden Gökdemir.

**Validation:** Olgu Aygün.

**Writing – original draft:** Olgu Aygün, Ayça Asma Sakalli.

**Writing – review & editing:** Özden Gökdemir.
